# Tumoral Malignancy Decreases Coupled with Higher ROS and Lipid Peroxidation in HCT116 Colon Cancer Cells upon Loss of PRDX6

**DOI:** 10.3390/antiox13070881

**Published:** 2024-07-22

**Authors:** Daniel J. Lagal, Antonio M. Montes-Osuna, Alberto Ortiz-Olivencia, Candela Arribas-Parejas, Ángel Ortiz-Alcántara, Cristina Pescuezo-Castillo, José Antonio Bárcena, Carmen Alicia Padilla, Raquel Requejo-Aguilar

**Affiliations:** 1 Department of Biochemistry and Molecular Biology, University of Córdoba, 14071 Córdoba, Spain; dlagal@health.ucsd.edu (D.J.L.); b92moosa@uco.es (A.M.M.-O.); b92orola@uco.es (A.O.-O.); b82arpac@uco.es (C.A.-P.); b72ortaa@uco.es (Á.O.-A.); b42pecac@uco.es (C.P.-C.); bb1barua@uco.es (J.A.B.); 2 Maimónides Biomedical Research Institute of Córdoba (IMIBIC), 14004 Córdoba, Spain

**Keywords:** cancer, ROS, peroxiredoxin 6, lipid peroxidation, malignancy

## Abstract

Peroxiredoxin 6 (PRDX6) is an atypical member of the peroxiredoxin family that presents not only peroxidase but also phospholipase A2 and lysophosphatidylcholine acyl transferase activities able to act on lipid hydroperoxides of cell membranes. It has been associated with the proliferation and invasive capacity of different tumoral cells including colorectal cancer cells, although the effect of its removal in these cells has not been yet studied. Here, using CRISPR/Cas9 technology, we constructed an HCT116 colorectal cancer cell line knockout for PRDX6 to study whether the mechanisms described for other cancer cells in terms of proliferation, migration, and invasiveness also apply in this tumoral cell line. HCT116 cells lacking PRDX6 showed increased ROS and lipid peroxidation, a decrease in the antioxidant response regulator NRF2, mitochondrial dysfunction, and increased sensitivity to ferroptosis. All these alterations lead to a decrease in proliferation, migration, and invasiveness in these cells. Furthermore, the reduced migratory and invasive capacity of HCT116 cancer cells is consistent with the observed cadherin switch and decrease in pro-invasive proteins such as MMPs. Therefore, the mechanism behind the effects of loss of PRDX6 in HCT116 cells could differ from that in HepG2 cells which is coherent with the fact that the correlation of PRDX6 expression with patient survival is different in hepatocellular carcinomas. Nonetheless, our results point to this protein as a good therapeutic target also for colorectal cancer.

## 1. Introduction

Colon cancer is the third most common cancer and the second leading cause of cancer-related deaths worldwide according to the World Health Organization. Despite the advances in early detection and therapeutic strategies, changes in human lifestyles and diet due to the global economy have increased the risk of colorectal cancer, making the development of new strategies and treatments against this disease crucial [1].

Changes in reactive oxygen species (ROS) homeostasis have been considered one of the main triggers of cancer progression and metastasis [2,3]. Tumor cells contain a higher amount of ROS than their normal progenitors [4]. However, the exact role of oxidative stress in cancer malignancy has not been established, due in part to both the beneficial and detrimental nature of ROS in tumor cells. The level of ROS-induced oxidative stress is higher in tumor cells and, while in non-tumor cells an increase in ROS and oxidative stress leads to induction of apoptosis, in tumor cells oxidative stress is more prone to act on cell signaling to maintain tumor phenotypes without signs of cytotoxic effects [5,6]. This is possibly due to an enhanced defense system based on an increase in antioxidant enzymes that protect cells from cell death mediated by large doses of ROS. In this context, the master regulator of antioxidant defense, the nuclear factor erythroid 2–related factor 2 (NRF2), is constitutively upregulated with some oncogenes triggering its activation [7,8]. NRF2 increases ROS tolerance and enhances the expression of numerous ROS scavengers such as superoxide dismutases (SODs), glutathione peroxidases (GPxs), and peroxiredoxins (PRDXs) [9,10].

PRDXs have been considered essential for maintaining redox homeostasis in all living organisms since around 90% of cellular peroxides are removed as a result of their catalytic capacity [11]. Depending on the specific PRDX family member and the cancer context, PRDXs can either inhibit cancer development or promote cancer growth [12]. PRDXs are characterized by the presence of a conserved Cys residue, called peroxidatic Cys, which is essential for their catalytic mechanism. In humans, there are six different PRDXs, classified in two subfamilies as 2-Cys (PRDX1-5) or 1-Cys (PRDX6) PRDXs based on the presence of a second reactive Cys residue called resolutive Cys. PRDX6 is the only member of the peroxiredoxin family with 1-Cys in its catalytic site and reduces H_2_O_2_, peroxynitrite, and hydroperoxides [13]. Furthermore, PRDX6 is also essential for membrane turnover; this enzyme can act on phospholipid hydroperoxides generated by lipid peroxidation [14], hydrolyses glycerophospholipids at the sn-2 position through its calcium-independent phospholipase A2 activity [15], and contributes to the transfer of fatty acyl CoA to the sn-2 position of lysophosphatidylcholine [16].

Besides the physiological role of PRDX6, this enzyme has been described as contributing to carcinogenesis by enhancing tumor growth and increasing invasiveness and metastasis in different tumors [17,18,19]. PRDX6 can favor ROS tolerance, contributing to cancer hallmarks and preventing ROS-derived cytotoxicity [20]. Our prior studies have shown that a lack of PRDX6 diminishes NRF2 levels in the SNU475 cell line and alters redox proteome in the HepG2 hepatocellular carcinoma cell line leading to decreased cell proliferation by repression of cell cycle progression [21,22]. Likewise, in the SNU-475 cell line, PRDX6 deficiency also decreases the migratory and invasive capacities of these cells [22]. Moreover, PRDX6, through its membrane turnover functions, can provide a deeper ROS tolerance, avoiding lipid peroxidation, which is essential to maintaining the proper organelle functionality and preventing ferroptosis in the presence of large ROS [23,24]. Indeed, PRDX6 downregulation is already considered a strategy to sensitize tumor cells to ferroptosis [24].

Despite the previous studies in hepatocarcinoma cells mentioned above, PRDX6 is a complex protein with dual effects due to its peroxidase and phospholipase A2 activities, so the effect of its deletion in different tumor cells cannot be extrapolated. This is reflected in the fact that PRDX6 levels correlate differently with patient prognosis depending on the tumor. This correlation differs for liver and colorectal tumors [25], making it relevant and novel to study for the first time the effect of PRDX6 deficiency in the colorectal cancer line HCT116. Thus, our results demonstrate a relevant aspect of PRDX6 in cancer cells. Despite some differences, the anti-proliferative and anti-migratory effects of its elimination are conserved between cancer cells suggesting that PRDX6 could be considered a key factor in different cell lines, in which its abolition would be a good therapeutic strategy.

## 2. Materials and Methods

Unless otherwise specified, all reagents were of analytical grade and purchased from Sigma-Aldrich (St. Louis, MO, USA). Cell culture dishes and flasks were from Techno Plastic Products AG (TPP). Trypsin and fetal bovine serum were acquired from Biowest (Nuaillé, France); McCoy’s medium was from Gibco (Grand Island, New York, NY, USA); antibodies against N-cadherin, E-cadherin, nuclear factor erythroid 2-related factor 2 (NRF2), and proliferating cell nuclear antigen (PCNA) were from Cell Signaling (Danvers, MA, USA); antibody anti-actin was from Santa Cruz Biotechnology (Dallas, TX, USA); and antibody anti-PRDX6 was from MyBiosource (Cambridge, UK). Secondary antibodies conjugated with Alexa Fluor 488 and 647 fluorophores were from Abcam (Cambridge, UK), and Matrigel^®^ Matrix basement membrane was from Corning (New York, NY, USA).

### 2.1. Cell Lines

HCT116 (CCL-247™) standard cell line was obtained from ATCC (American Type Culture Collection, Manassas, VA, USA), used for between 5 and 20 passages, and cultured in McCoy´s medium (Gibco). The medium was supplemented with 10% fetal bovine serum (FBS), 100 U/L penicillin, 100 g/mL streptomycin, and 0.25 g/mL amphotericin (Gibco). Cells were grown in aseptic conditions at pH 7.4 in an incubator with a 5% CO_2_ atmosphere and at 37 °C. The cell line was validated and tested for mycoplasma every three months.

### 2.2. Construction of an HCT116 Cell Line without Peroxiredoxin 6 (HCT116^PRDX6−/−^) Using the CRISPR/Cas9 Methodology

HCT116 cell line knockout for PRDX6 was generated by applying the CRISPR/Cas9 technique twice using the same protocol and specific gRNA set-up for HepG2 and SNU475 cell lines [21,22]. A total of 6 × 10^4^ cells/cm^2^ were cultured in 24 well plates and transfection was carried out for 72 h. Then, cells were detached, and a fraction was used to calculate the efficiency. The rest of the cells were grown individually, and the clones were analyzed for PRDX6 by Western blot with specific antibodies.

### 2.3. Extracellular Flux Analysis of Mitochondrial Respiration and Glycolytic Function

Mitochondrial respiration using Agilent Seahorse XF24 Analyzer and Agilent Seahorse XF Cell Mito Stress Test (Agilent Seahorse Bioscience, Santa Clara, CA, USA) was assayed as previously described [21]. At 48 h before the assay, HCT116 cells were seeded at 4 × 10^4^ cells per well in a Seahorse 24-well XF Cell Culture microplate. Mitochondrial function was analyzed by sequential injections of the modulators oligomycin (1 µM), carbonyl cyanide-p-trifluoromethoxyphenylhydrazone (FCCP, 0.25 µM), and rotenone/A (0.5 µM). The oxygen consumption rate (OCR) was measured at the beginning and after adding each modulator and the basal, ATP production-linked, maximal, proton leak-linked OCR, spare respiratory capacity, and non-mitochondrial respiration parameters determined following the manufacturer´s guidelines. Data were normalized to protein concentration determined at the end of the assay.

### 2.4. Measurement of Enzymatic Activities, ROS, Lipid Peroxidation, Ferroptosis, and Protein

Activity of the mitochondrial complexes I/III was measured in 20 mM KPi buffer and 0.1 mM ethylenediaminetetraacetic acid (EDTA), at pH 8.0 and containing 2 mM potassium cyanide (KCN). After equilibration for 10 min, cytochrome c, 50 μM, was added, and its reduction was determined at 550 nm. After that, the activity not corresponding to complex I was assayed after adding 10 μM of rotenone [26]. Reactive oxygen species (ROS) were measured, following the manufacturer’s instructions, using a commercial ROS assay kit (Canvax Biotech S.L., Córdoba, Spain). Lipid peroxidation was quantified using a thiobarbituric acid reactive substances (TBARS) assay kit (Canvax Biotech S.L.), based on the reaction of malondialdehyde (MDA) derived from peroxidized lipids with thiobarbituric acid (TBA). Ferroptosis was evaluated by cell viability assay using an XTT Cell Proliferation Assay Kit (Canvax Biotech S.L.) and erastin (0–20 μM) and ferrostatin (2 μM) as inductor and inhibitor, respectively. Protein concentration was determined using a bicinchoninic acid (BCA) assay kit (Thermo Fisher Scientist, Waltham, MA, USA) with bovine serum albumin (BSA) as the standard.

### 2.5. SDS-PAGE and Western Blotting

SDS-PAGE for the detection of specific proteins and oxidative damage was performed in 10–15% gels. After electrophoresis, proteins were transferred to a nitrocellulose membrane in a semi-dry electrophoretic transfer system (Bio-Rad Laboratories; Hercules, CA, USA) for 45 min at 400 mA or by wet transfer for 1 h and 15 min at 120 V. Transfer and protein load were checked by staining with Ponceau reagent. The membranes were blocked for 1 h and incubated overnight at 4 °C with the corresponding dilutions of primary antibodies (1:2000, PRDX6; 1:200, glutaredoxin 1 (Grx1); 1:1000, NRF2, complex I, PCNA, E-cadherin and N-cadherin; and 1:4000, actin). They were then washed with 1× Tris-buffered saline, 0.1% Tween^®^ 20 detergent (TBS-T), and incubated with the corresponding peroxidase-conjugated secondary antibody (1:4000 anti-rabbit, anti-goat; or 1:8000 anti-mouse). The chemiluminescent signal induced by ECL reagent (GE Healthcare) was detected in a ChemiDoc image analyzer (Bio-Rad Laboratories; Hercules, CA, USA) and quantified by densitometry with ImageJ software version 1.51 (National Institutes of Health, Bethesda, MD, USA), using actin as a reference for loading normalization.

### 2.6. Transmission Electron Microscopy

Cells were detached, centrifuged, and fixed for 30 min in 2.5% glutaraldehyde prepared in 0.1 mol/L phosphate buffer (pH 7.4). Then, cells were post-fixed in 1% osmium tetraoxide in the same buffer for 30 min, dehydrated in graded ethanol, washed with propylene oxide, embedded in Epon, and then sectioned on an ultramicrotome at 90 nm thickness. Sections were stained with 5% uranyl acetate and 5% lead citrate and then analyzed on a JEM1400 (Japan Electron Optics Laboratory, Tokyo, Japan) transmission electron microscope at 80 kV.

### 2.7. Cell Proliferation and Cell Cycle Analysis

HCT116^PRDX6+/+^ and HCT116^PRDX6−/−^ cells (1.5 × 10^4^) were seeded in 24-well plates and allowed to grow for 1–4 days. Cells were then collected and counted generating the corresponding growth curves. Cell proliferation was also analyzed by XTT assay and viability by colony-forming unit (CFU) assay as previously described [27]. For cell cycle analysis, 6.5 × 10^5^ of these cells were seeded in 6-well plates and incubated for 24 h. After that, a 3 h pulse with bromodeoxyuridine (BrdU), 10 μM, was carried out for BrdU incorporation, and cell proliferation was determined by flow cytometry using the APC BrdU Flow Kit (Becton Dickinson Biosciences, Franklin Lakes, NJ, USA) and the cytometer BD AccuriTM C6 Plus (BD Biosciences). The proportion of cells in different cycle phases was established from 7-AAD-stained cells. Data were processed with BD Accuri™ C6 Plus 1.0.34 software (BD Biosciences).

### 2.8. Metalloproteinase Activity Detection Assay

Wild-type and knockout for PRDX6 HCT116 cells (3 × 10^5^) were seeded in 6-well plates and grown for 48 h in McCoy´s complete medium. After that, the medium was changed, and the cells were maintained in FBS-free medium for an additional 48 h. After that time, the cell culture medium was collected and cells were scraped in lysis buffer and both were used for metalloproteinase activity determination (zymogram) as described [25].

### 2.9. Wound Healing Assay

Cell migration was evaluated by a wound scratch assay as previously described [22] with some modifications. HCT116^PRDX6+/+^ or HCT116^PRDX6−/−^ cells were seeded at a concentration of 2 × 10^4^ cells/well in 24-well plates. When cells reached a confluence of 90%, each well was artificially wounded by creating a scratch with a plastic pipette 100 μL tip on the cell monolayer. Cell migration progressed for 20 h and wound closure was measured after crystal violet staining quantifying the free-cell area.

### 2.10. Transwell Invasion Assay

HCT116P^RDX6+/+^ or HCT116^PRDX6−/−^ cells were seeded at a density of 2.5 × 10^4^ cells in transwell chambers coated with Matrigel as described [22]. The cells were allowed to invade for 24 h and after fixing and staining with crystal violet were analyzed with a Leica optical microscope (Wetzlar, Germany).

### 2.11. Statistics

Statistical analysis of the data was performed using the Mann–Whitney test, unpaired Student’s *t*-test, and one or two-way ANOVA, depending on the experiment design, as detailed in the figure legends. The data are expressed as mean ± SD of at least three independent experiments in triplicate and the threshold for statistically significant differences is set at a *p*-adjusted < 0.05 value.

## 3. Results

### 3.1. Construction of a Colon Cancer Cell Line Lacking PRDX6

**Figure 1:**
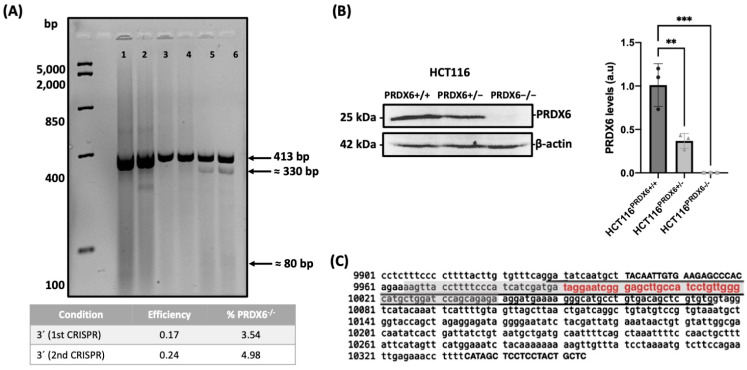
Construction of a stable HCT116^PRDX6−/−^ cell line. (**A**) Efficiency and probability of knockout HCT116 cells of consecutive CRISPR-Cas9 procedures. Lanes 1, 2 correspond to the original PCR amplification product; lanes 3, 4 negative controls without endonuclease; and lanes 5, 6 cleavage produced by endonuclease T7 in the 1st and 2nd CRISPR, respectively. (**B**) Analysis of clones by Western blot normalized and relative to HCT116^PRDX6+/+^ (one-way ANOVA, *p*-value, n = 3 ** ≤ 0.01 *** *p*-value ≤ 0.001). (**C**) Sequence of prdx6 gene with exon 3 underlined, gRNA in red color, primers in capital letters, and region containing deletions in knockout cells dark shaded.

The HCT116 knockout cell line for PRDX6 (HCT116^PRDX6−/−^) was constructed using the same methodology and sgRNA described [21]. The cells were transfected using the CRISPR-Cas9 technique and the efficiency was calculated obtaining a value of 0.17 and a percentage of transformed cells of 3.54% (Figure 1A). These cells were used to carry out a limit dilution in 96-well plates, and the individual clones analyzed for the presence of PRDX6 by Western blot with specific anti-PRDX6 antibodies. Some clones were knocked down for PRDX6 by 50% but none of them were knocked-out for PRDX6 meaning a successful knockout for only one allele of *prdx6* gen (Figure 1B). Thus, a second CRISPR was performed using one of the clones with a lower expression of PRDX6 obtaining an efficiency of 0.24 and a percentage of transformed cells close to 5% (Figure 1A). A second limit dilution was carried out and the clones analyzed by Western blot resulting in one of them being fully knocked-out for PRDX6 (Figure 1B). Sanger sequencing of the clone demonstrated that disruption of the *prdx6* gene was biallelic with deletions of 22 and 14 base pairs in each allele surrounding the zone complementary to the gRNA (Figure 1C).

### 3.2. Lack of PRDX6 in HCT116 Cells Induces Oxidative Stress and Lipid Peroxidation Sensitizing Cells to Ferroptosis

**Figure 2:**
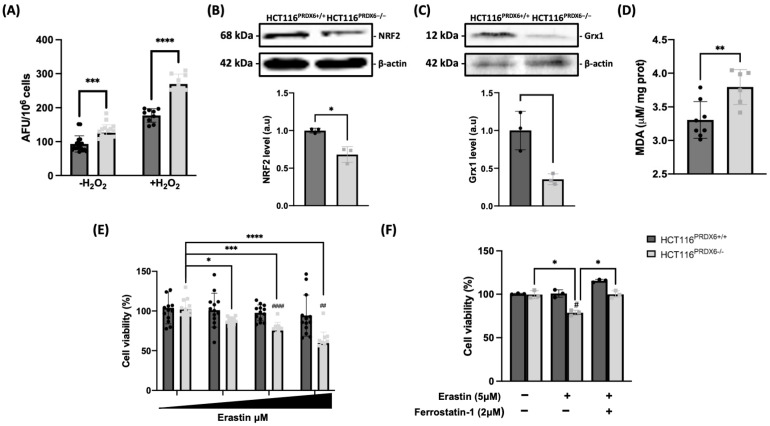
Role of PRDX6 on oxidative stress parameters in HCT116 cell lines. (**A**) ROS levels in basal and H2O2 50 μM treated cells expressed as arbitrary fluorescence units (AFU) per million cells in the 2′,7′ –dichlorofluorescin diacetate (DCFDA) assay. (**B**) Nuclear factor erythroid 2-related factor 2 (NRF2) protein levels measured by Western blot and expressed as arbitrary units (a.u.) normalized to β-actin and relative to HCT116^PRDX6+/+^. (**C**) Glutaredoxin1 (Grx1) levels determined by Western blot normalized and relative to HCT116^PRDX6+/+^, expressed as arbitrary units. (**D**) Lipid peroxidation determined by thiobarbituric acid reactive substance assay (TBARS) measuring malondialdehyde (MDA) production and normalized by protein concentration. (**E**) Ferroptosis measured as cell viability in HCT116^PRDX6+/+^ and HCT116^PRDX6−/−^ cells treated with erastin (0, 5, 10, and 20 μM) for 72 h; * indicates differences between erastin treated cells and # indicates differences between HCT116^PRDX6+/+^ and HCT116^PRDX6−/−^ cells. (**F**) Ferroptosis of cells treated with erastin in the absence or presence of ferrostatin-1 for 24 h. Black circles and grey squares are the individual values of the different replicas of HCT116^PRDX6+/+^ and HCT116^PRDX6−/−^ cells, respectively; * indicates differences between erastin/ferrostatin-1 treated cells and # indicates differences between HCT116^PRDX6+/+^ and HCT116^PRDX6−/−^ cells. Student’s *t*-test for single comparisons and ordinary one-way ANOVA for ferroptosis assays were performed. (N = 3, n ≥ 3, * # *p*-value ≤ 0.05; ** ## *p*-value ≤ 0.01; *** *p*-value ≤ 0.001; **** #### *p*-value ≤ 0.0001).

The level of ROS was determined in wild-type and knockouts for PRDX6 HCT116 cells treated with H_2_O_2_, proving to be higher in cells lacking PRDX6 and further increasing in the presence of H_2_O_2_ (Figure 2A). Surprisingly, as has been observed in other tumoral cell lines [28], the levels of the antioxidant master NRF2 (Figure 2B) and non-NRF2-dependent antioxidant proteins such as glutaredoxin 1 (Grx1) (Figure 2C), were also lower in HCT116^PRDX6−/−^ cells contributing even more to the increase in oxidative stress. The increase in ROS in HCT116^PRDX6−/−^ cells was accompanied by lipid peroxidation and lower cell viability related to ferroptosis as evidenced by erastin sensitivity and recovery of viability with ferrostatin-1 (Figure 2D–F). This would explain the higher number of lipid droplets observed in HCT116^PRDX6−/−^ cells by electron microscopy, which should be due to an attempt by these cells to avoid ferroptosis by sequestrating lipid peroxides.

### 3.3. Severe Impairment of Mitochondrial Function and Biogenesis Occurs in HCT116 Cells When PRDX6 Is Removed

**Figure 3:**
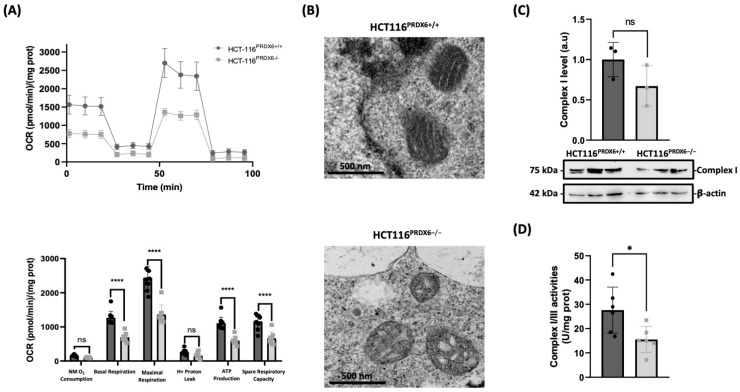
Mitochondrial function and structural analysis of PRDX6-deficient HCT116 cell line. (**A**) Representative recording of oxygen consumption rate (OCR) during extracellular flow analysis for the Mito Stress Test and histogram plots of the calculated non-mitochondrial (NM) O_2_ consumption, basal and maximum respiratory rate, H^+^ proton leak, ATP production rate, and spare respiratory capacity, all normalized for protein content. (**B**) Mitochondrial visualization by transmission electron microscopy (TEM) of HCT116^PRDX6+/+^ and HCT116^PRDX6−/−^. (**C**) Complex I (75 KDa) protein determination by Western blot relative to wild-type, normalized by β-actin and expressed as arbitrary units (a.u). (**D**) Complex I and III enzymatic activities normalized for protein content. (N = 3, n ≥ 3, Multiple *t*-test, * *p*-value ≤ 0.05; **** *p*-value ≤ 0.0001; ns not significant).

Given the increased oxidative stress in HCT116^PRDX6−/−^ cells, mitochondrial function was tested next. As expected, mitochondrial functionality was decreased in cells lacking PRDX6. Parameters such as basal and maximum respiration, and spare respiratory capacity, were lower in HCT116^PRDX6−/−^ cells, decreasing by approximately 50% (Figure 3A). This agreed with the changes observed in the morphology of the mitochondria of these cells, which was altered showing internal disorganization of the mitochondrial cristae (Figure 3B). Although no differences were observed in complex I protein level (Figure 3C), the marked decrease in complex I/III activities (Figure 3D), probably due to their susceptibility to oxidative stress, should be behind the respiratory incapacity of HCT116^PRDX6−/−^ cells.

### 3.4. Lack of PRDX6 in HCT116 Colorectal Cancer Cells Causes Decreased Cell Proliferation without Changes in Cell Viability

**Figure 4:**
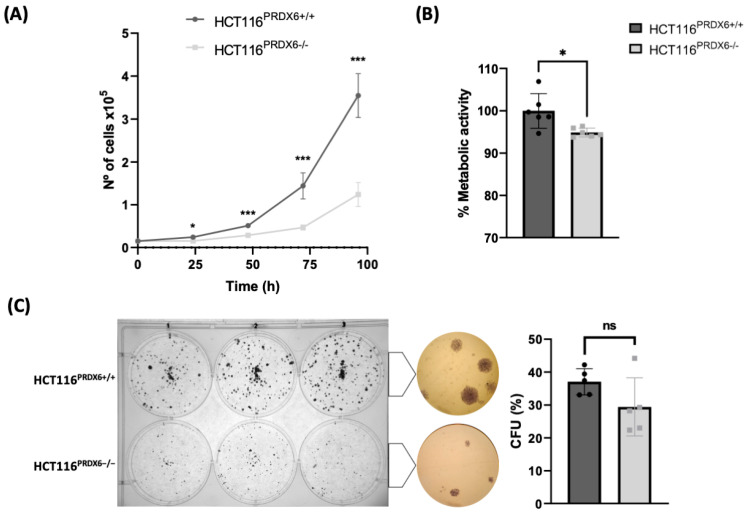
Effect of PRDX6 on growth and cell viability of HCT116 tumor cells. (**A**) Growth curve showing the number of cells after 24, 48, 72, and 96 h of cell culture. (**B**) Cell viability determined using XTT assay and expressed as arbitrary units (a.u). (**C**) Representative colony forming units (CFU) assay (left) and rate of colonies (right) after 10 days of cell culture. Data were obtained by colony counting with ImageJ software version 1.51. (N = 3, n ≥ 3, *t*-test, * *p*-value ≤ 0.05; *** *p*-value ≤ 0.001; ns not significant).

Cell proliferation determined by cell growth curves and XTT assay indicated a lower proliferation rate of HCT116^PRDX6−/−^ cells compared with HCT116^PRDX6+/+^ (Figure 4A,B). This decrease in cell number was not due to increased apoptosis since no sign of cell death was observed and the viability of HCT116^PRDX6−/−^ and HCT116^PRDX6+/+^ cells was similar (Figure 4C). The colony-forming unit (CFU) assay showed that although the number of colonies did not differ, their size was markedly larger in wild-type cells, consistent with the greater proliferation of these cells.

### 3.5. Absence of PRDX6 in HCT116 Cells Triggers Cell Cycle Arrest in G2/M

**Figure 5:**
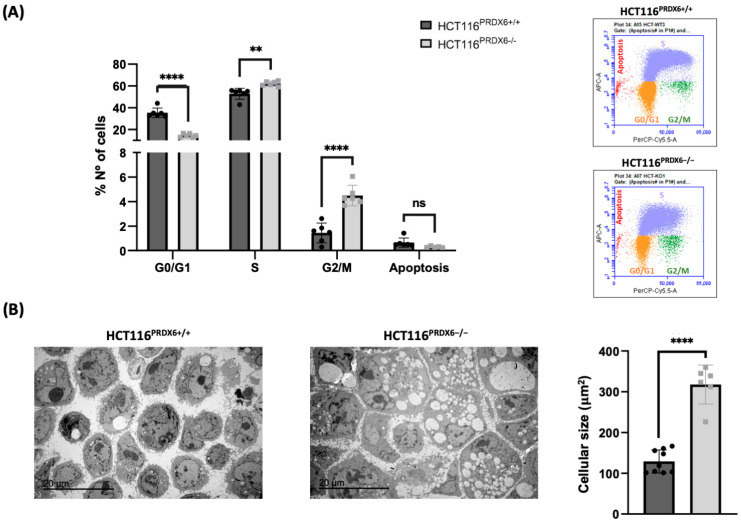
Cell cycle parameters of HCT116^PRDX6+/+^ and HCT116^PRDX6−/−^ cell lines. (**A**) Flow cytometry analysis showing the distribution of cells along the different cell cycle phases and those undergoing apoptosis (left); the recordings of cytometer fluorescence event counts of one representative experiment are shown (right); red indicates apoptotic cells; orange, G0/G1 phase; blue, S phase; and green, G2/M phase. (**B**) Cell visualization by transmission electron microscopy (TEM) (left), and cell size determination (right), the cellular area was measured using ImageJ software. (N = 3, n ≥ 3, multiple *t*-test, ** *p*-value ≤ 0.01; **** *p*-value ≤ 0.0001; ns not significant).

Proliferation was also measured as bromodeoxyuridine (BrdU) incorporation into DNA by flow cytometry, but, in this case, the results indicate a significantly higher amount of HCT116^PRDX6−/−^ cells in the S phase compared to normal HCT116^PRDX6+/+^ cells (Figure 5A), contrary to the decrease in cell proliferation observed in the growth curves and XTT assay (Figure 4A,B). This can be explained by the distribution of these cells in the different phases of the cell cycle since an arrest of HCT116^PRDX6−/−^ cells at the G2/M phase was observed (Figure 5A). Interestingly, the size of these cells is larger than the normal HCT116^PRDX6+/+^ cells (Figure 5B), consistent with cell cycle arrest at G2/M and less proliferation. To explain this arrest, we focused on the proliferating cell nuclear antigen (PCNA), whose expression level and redox state have been described to be altered by the lack of PRDX6 in other tumor cell lines [21,22]. However, the level of PCNA did not change in HCT116^PRDX6−/−^ cells, and it is not the cause of the cell cycle arrest in these cells.

### 3.6. Colorectal Cancer Cells Lacking PRDX6 Exhibit More Epithelial Features, Leading to a Reduced Capacity for Migration and Invasion

**Figure 6:**
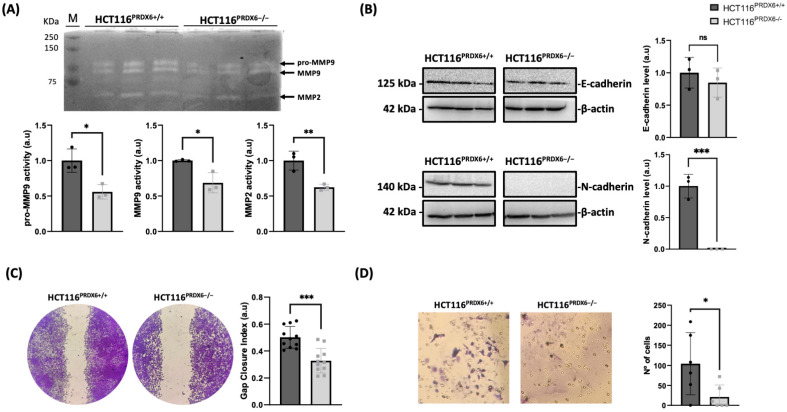
Epithelial–mesenchymal transition (EMT), migration, and invasiveness of HCT116^PRDX6+/+^ and HCT116^PRDX6−/−^ tumor cells. (**A**) Enzymatic activity of pro-MMP9 (92 kDa), MMP9 (82 kDa), and MMP2 (64 kDa) on a gel containing 0.08% gelatin; each lane represents 20 µg of protein from medium (extracellular) of both cell lines. (**B**) E-cadherin and N-cadherin levels determined by Western blot relative to wild-type cells and normalized by β-actin. (**C**) Representative wound healing assay with cells visualized after 20 h of making the gap (left) and quantitation of the gap closure index (GCI) (right). (**D**) Representative Matrigel^®^ transwell cell invasion assay using the same number of cells at time 0 and the endpoint at 24 h, showing fixed and stained translocated invasive cells. Bright-field images under 10× magnification were taken and the number of cells quantified. (N = 3, n ≥ 3, *t*-test, * *p*-value ≤ 0.05; ** *p*-value ≤ 0.01; *** *p*-value ≤ 0.001; ns not significant). (MMP9: metalloproteinase 9; MMP2: metalloproteinase 2).

To determine the effect of PRDX6 deficiency not only in proliferation but also on the metastatic capacity of HCT116 cells, parameters related to epithelial–mesenchymal transition (EMT), and the migratory and invasive capacity of these cells were evaluated. Metalloproteinases (MMPs) play a key role in tumor angiogenesis and metastasis. Therefore, we determined how MMP2 and MMP9 were affected when PRDX6 was removed. Both MMP2 and MMP9 showed a lower secretion into the culture medium, which may be related to reduced migration and invasion capacity (Figure 6A). As epithelial and mesenchymal markers, E-cadherin and N-cadherin levels were analyzed. E-cadherin showed no difference between HCT116^PRDX6−/−^ and HCT116^PRDX6+/+^ cells. However, N-cadherin was not detected in HCT116^PRDX6−/−^ cells, indicating a more epithelial phenotype of these cells when PRDX6 is removed (Figure 6B). Finally, migration and invasion were analyzed. HCT116^PRDX6−/−^ cells were less able to migrate, as can be observed by the reduced ability to close the gap in the wound healing assay (Figure 6C), and to invade; the invasion assay showed a decrease in the number of cells passing from an upper to a lower chamber when PRDX6 was absent (Figure 6D). All these findings suggest a role of PRDX6 in HCT116 dedifferentiation characterized by high levels of N-cadherin and increased levels and activity of metalloproteinases which could contribute to the metastatic features of colorectal cancer cells.

## 4. Discussion

Peroxiredoxins (PRDXs) are multifunctional enzymes upregulated in cancer cells that act to enhance tumor growth, metastasis, and drug resistance [29]. Thus, the downregulation of peroxiredoxin activities through inhibitors or knocking down their expression has been considered a promising strategy for cancer treatment. However, this strategy can cause undesirable side effects, as shown by the fact that animal models lacking PRDXs _(1-5)_ present anomalies such as severe anemia, insulin resistance, testicular atrophy, steatosis, and, in the case of PRDX1, cancer development and a shortened life span [30]. Nevertheless, animal models with knockout for PRDX6 are viable, fertile, and show no morphological defects. In addition, deletion of PRDX6 in hepatocarcinoma lines did not increase the remaining PRDXs [21,22] in what could be a compensatory mechanism. Therefore, PRDX6 could be the better choice as a target within the family of PRDXs since its removal may affect tumor cells with a minimal effect on normal ones. The effect of PRDX6 loss has been studied in two tumoral hepatocarcinoma cell lines [21,22] with different degrees of differentiation, HepG2 and SNU475, but, still, it is unknown whether it can be extrapolated to other tumors including human colorectal carcinoma. For this purpose, we constructed a monoclonal colon cancer cell line HCT116 knockout for PRDX6 using CRISPR-Cas9 technology that generated deletions in both alleles of *prdx6* gen. As expected, PRDX6 removal increased reactive oxygen species (ROS) probably due to the loss of its peroxidase activity and the decreased expression of the transcription factor nuclear factor erythroid 2-related factor 2 (NRF2) and the NRF2-independent antioxidant protein glutaredoxin1 (Grx1), in agreement with previous observations made in hepatocellular carcinoma cell lines [21,22]. Increased ROS leads to lipid peroxidation in PRDX6 knockout cells. PRDX6 has a crucial role in the reversion of lipid peroxidation under mild–moderate oxidative stress, contributing to the repair of peroxidized cell membranes. Thus, phospholipid hydroperoxides are reduced by PRDX6 through its peroxidase activity or removed by hydrolysis and re-acylation with a fatty acyl CoA through its phospholipase A2 (PLA_2_) and lysophosphatidylcholine acyl transferase activities [14,15], and a lack of PRDX6 may lead to altered levels of lipids involved in signaling pathways and to an increase in lipid peroxidation. Lipid peroxidation has been described as the main trigger of ferroptosis, an iron-dependent mechanism of non-apoptotic cell death [31]. Our results indicate an increased susceptibility of HCT116 knockout cells to erastin-induced ferroptosis, which was reversed by ferrostatin-1 treatment. Several studies have identified PRDX6 as a negative regulator of ferroptosis. The silencing of PRDX6 in epithelial and tumor cell lines such as H1299, A549, and 293FT and lung cancer cells significantly enhanced ferroptosis induced by erastin. At the same time, its upregulation in podocytes prevents ferroptosis and renal glomerular damage [24,32,33]. Induction of ferroptosis has been considered an effective strategy for tumor therapy [34], hence, PRDX6 silencing might be a good therapeutic option to enhance ferroptosis and diminish the viability of colon cancer cells.

It has been well documented that PRDX6 protects against mitochondrial dysfunction by alleviating the loss of mitochondrial membrane potential in several pathologies [21,22,23,35]. PRDX6 is also the only member of the PRDX family that translocates to damaged mitochondria [36]. Therefore, it is not surprising that HCT116^PRDX6−/−^ showed a decreased respiration rate and a diminished respiratory capacity, disruption of mitochondrial membranes, and inactivity of mitochondrial complexes I/III. Loss of both peroxidase and PLA_2_ activities of PRDX6 may contribute to mitochondrial damage through increased ROS with damage of mitochondrial components and failure to repair cell membranes subjected to phospholipid peroxidation [37].

The lack of PRDX6 also resulted in reduced cell proliferation and cell cycle arrest, as has been described in other tumoral and non-tumoral cell lines [21,22,38]. A decrease in PLA_2_ levels of PRDX6 can lead to altered levels of lipids and related downstream signaling pathways, while a decrease in peroxidase activity can produce redox changes in key proteins. Lipid metabolism is widely altered in cancer cells that present increased lipogenesis, fatty acid uptake, and fatty acid oxidation for membrane synthesis, energy production, and signaling that support cell proliferation. However, dysregulated lipid metabolism with an increase in lipid saturation and oxidation can lead to apoptotic and ferroptotic cell death, respectively [39,40]. Likewise, the proliferation, invasion, and metastatic capacity of tumor cells are highly regulated and dependent on their protein redox state [41]. Among the cell cycle-related proteins that modified their expression in the absence of PRDX6 we found proliferating cell nuclear antigen (PCNA). PCNA is an essential factor in cell cycle progression with high expression in cancer cells [42]. Reduced levels of this protein have been related to a decrease in the proliferative, migratory, and invasive capacities of lung and prostate cancer cells [43,44]. A decrease in PCNA activity has also been associated with cell cycle arrest at S and G2/M phases [45] in hepatocarcinoma cell lines. The arrest could be generated by a decrease in the level of the protein and oxidation of critical amino acid residues [21,22]. HCT116^PRDX6−/−^ cells showed no change in PCNA levels but a study of PCNA oxidation state should be conducted to elucidate the possible relationship of this protein redox regulation [21] to cell cycle arrest at the G2/M phase, which could explain the significantly larger size of these cells. Cell cycle arrest could also be favored by the absence of PRDX6 phospholipase A_2_ activity and the associated changes produced on cell lipids. We observed a huge increase in lipid droplets in cells lacking PRDX6. Lipid droplet formation in cell cycle-arrested cells has been described as a mechanism to sequestrate lipid peroxides and polyunsaturated fatty acids protecting these cells from ferroptosis [46].

One of the main limitations of PRDX6 deletion is that although it reduces proliferation through cell cycle arrest, it does not seem to affect the viability of tumor cells. However, it has been described that PRDX6 is associated with resistance to chemotherapy [12], so an effective way of reducing viability may be to use chemotherapy coupled with the removal of this protein.

Changes in the lipid content of cancer cells are also related to their invasion and metastatic capacity [47]. These changes may be mediated by both PLA_2_ and peroxidase activities of PRDX6. Several studies indicate that overexpression of PRDX6 induces migration and invasion and promotes EMT transition while its silencing inhibits them [22,25,48,49,50,51]. We observed that complete removal of PRDX6 markedly decreased the migration and invasiveness of HCT116 cells and that this was accompanied by diminished secretion of metalloproteinases and a total absence of N-cadherin pointing to a more epithelial phenotype in these cells which agrees with previous studies. This may be due to alterations in signaling pathways such as phosphatidylinositol 3-kinase/protein kinase B (PI3K/Akt), p38 and extracellular signal-regulated kinases 1/2 (Erk1/2) [48,49,52], and the rearrangement of the cell membranes due to lipid alteration, causing changes in the cytoskeleton, size, motility, and metalloproteinases secretion of HCT116^PRDX6−/−^ cells as described for the mesenchymal hepatocarcinoma cell line SNU475 [22].

In summary, the absence of PRDX6 in colon cancer cells increases ROS and oxidative damage in the form of lipid peroxides. The increase in lipid peroxidation and the alteration of the lipid content of HCT116^PRDX6−/−^ cells due to the loss of both peroxidase and PLA_2_ activities leads to a greater susceptibility to ferroptosis, cell membrane damage, mitochondrial dysfunction, and cell cycle arrest, which are exacerbated by decreased expression of proteins such as NRF2 and Grx1. This results in reduced proliferation, migration, and invasion of HCT116^PRDX6−/−^ cells as occurs in other tumor cells thus confirming PRDX6 as a general target for the treatment of these malignancies. The challenge is to block Prdx6 expression in tumor cells without affecting normal cells. Fortunately, gene silencing and genetic engineering techniques are progressing enormously [53], and new targeted therapy tools are being developed to treat cancer, which will surely allow us to achieve this goal in the near future.

## Data Availability

Data sharing does not apply to this article as no datasets were generated or analysed during the current study. However, data corresponding to figures and replicates of the experiments shown in the article (e.g., Western blots) will be available as requested.
